# Review of MDA registers for Lymphatic Filariasis: Findings, and potential uses in addressing the endgame elimination challenges

**DOI:** 10.1371/journal.pntd.0008306

**Published:** 2020-05-14

**Authors:** Dziedzom K. de Souza, Katherine Gass, Joseph Otchere, Ye Min Htet, Odame Asiedu, Benjamin Marfo, Nana-Kwadwo Biritwum, Daniel A. Boakye, Collins S. Ahorlu

**Affiliations:** 1 Department of Parasitology, Noguchi Memorial Institute for Medical Research, College of Health Sciences, University of Ghana, Legon-Accra, Ghana; 2 Task Force for Global Health, Decatur, Georgia, United States of America; 3 Emory University, Atlanta, Georgia, United States of America; 4 Neglected Tropical Diseases Programme, Ghana Health Service, Accra, Ghana; 5 Bill and Melinda Gates Foundation, Seattle, Washington, United States of America; 6 Department of Epidemiology, Noguchi Memorial Institute for Medical Research, College of Health Sciences, University of Ghana, Legon-Accra, Ghana; University of Buea, CAMEROON

## Abstract

**Background:**

Lymphatic filariasis (LF) is endemic in Ghana, and the country has implemented the GPELF strategy since 2000 with significant progress made in the control of the disease. However, after several years of mass drug administration (MDA) implementation, there is persistent transmission in 17 of the 98 endemic districts in the country. Current approaches to surveillance are clearly unable to target untreated individuals and new strategies are required to address the endgame challenges to enhance LF elimination as a public health problem in endemic countries. Community registers are used during MDAs to enumerate community members, their age, gender, house numbers, and records of their participation in MDAs. These MDA registers represent an untapped opportunity to identify and characterize non-compliance and inform appropriate programmatic actions. In this study, we analyzed the data presented in the registers to assess the coverage and individuals’ compliance in MDA.

**Methods:**

The information in the MDA registers were assessed to verify the reported coverages obtained from the district. The community registers were obtained from the district health offices and the data from each individual record was entered into a database. A simple questionnaire was used to cross-check the participation of study participants in the 2017 MDA. The questionnaire solicited data on: participation in the 2017 MDA, reasons for not taking part in the MDA, adverse events experienced, what was done for the adverse events, and willingness to participate in subsequent MDAs.

**Results:**

We found that 40.1% of the population in the registers missed at least one MDA in 3 years (2016–2018) and the majority of them were between 10–30 years of age. The results of the questionnaire assessment indicated that 13.8% of the respondents did not receive treatment in 2017 for various reasons, the most prominent among them being “absence/travel” (37.1%). Data in the registers were used to verify the treatment coverage for the years 2017 and 2018, and reviewed against the reported coverage obtained from the district. Significant differences between the reported and verified coverages were only observed in four communities. However, the assessment also revealed that the reported coverage was only accurate in 33.3% of cases.

**Conclusions:**

The MDA registers allow for the identification of eligible individuals who were not reached during any MDA round. Thus, the MDA registers could be utilized at the community and programme levels to identify missing and untreated individuals, appropriately address their non-compliance to MDA, and thereby improve MDA coverage in each implementation unit and monitor the progress towards elimination of LF. The challenges observed through the review of the registers also offer opportunities to improve the training given to the community drug distributors.

## Author summary

Elephantiasis is a disease that is common in Ghana. Since the year 2000 the country has implemented a control programme aimed at eliminating the disease as a public health problem, through the yearly treatment of entire communities in districts where the disease is found. Achievements have been made, and treatment has stopped in many districts; however, some districts have received many years of treatment without successfully reducing the infections below the targeted levels. Many individuals go untreated every year, as can be observed from the low reported treatment coverage after MDA. This untreated population may explain why some districts have failed to achieve the criteria for stopping treatment. Fortunately, the treatments are recorded in community registers, which contain the details of all community members, as well as their treatment participation. These registers can therefore be used to identify people who have not received treatment and design appropriate interventions to reach them. Through these registers, we were able to identify and characterize individuals who were not treated between 2016 and 2018 in 10 communities. We found that 4 out of 10 people missed at least one treatment between 2016 and 2018, and the majority of them were 10–30 years of age. Using the information from the registers we were also able to verify the proportion of people who took part in the treatments in 2017 and 2018, and compared this to what was reported. This comparison revealed that the reporting was only accurate in 33% of cases. A questionnaire was also used to cross-check the participation of study participants in the 2017 MDA, with results indicating that 13.8% of the respondents did not receive treatment for various reasons, the most prominent among them being “absence/travel” (37.1%). Although these registers are seldom used by the Lymphatic Filariasis Control Programme for anything other than recording and reporting treatment information, this study shows that the registers provide an opportunity to identify individuals who have not received treatment, develop a plan to appropriately address their reasons for not taking part in the treatments, target these untreated individuals to improve the overall proportion of people taking part in MDA, and assess the accuracy of reported coverage information. These activities will allow the programme to better monitor the progress towards, and ultimately achieve, elimination of lymphatic filariasis as a public health problem.

## Introduction

Lymphatic filariasis (LF) is a tropical parasitic disease caused by one of three filarial nematodes, with over 90% of cases attributed to *Wuchereria bancrofti*. Depending on the geographic region, it is transmitted by mosquitoes belonging to the *Anopheles*, *Culex*, *Aedes* and *Mansonia* genera. While majority of infected individuals are asymptomatic, prolonged untreated infection over several years may result in lymphedema of the limbs in both males and females, and hydrocele in males [1]. This leads to disfigurement and eventual disability, affecting the ability to work, reduced access to services and social inclusion of affected individuals, and often with devastating economic and mental health consequences for affected individuals [2]. Thus, it is considered the world’s second most disabling condition, after mental-illness [3].

LF is one of the neglected tropical diseases (NTDs) targeted for elimination by the World Health Organization (WHO) as a public health problem. In the year 2000, the Global Programme to Eliminate Lymphatic Filariasis (GPELF) was set up, following the adoption of the World Health Assembly resolution WHA50.29 in 1997 calling WHO member states to eliminate LF. The goal of the GPELF was to eliminate the disease by the year 2020 through two principal strategies: i) interrupting the transmission of the disease through the yearly treatment of entire endemic communities [4, 5] and ii.) alleviating the suffering resulting from LF morbidity and disability [6]. The treatment of endemic communities is based on the combination treatment of albendazole together with ivermectin and or diethylcarbamazine, depending on the geographic setting and co-endemicity with other diseases such as onchocerciasis and loa loa [7]. A coverage of at least 65% of the endemic population is recommended for a minimum of 5 years, in order to reduce infection to levels below which transmission can no longer be sustained [8]. By the end of 2017, more than 7.1 billion treatments had been delivered to more than 890 million people, at least once [1]. Out of 72 endemic countries, 11 (2 in Africa) have been validated as having eliminated LF as a public health problem, 10 countries stopped mass drug administration (MDA) and are under surveillance, 46 are implementing MDA and 5 are yet to start MDA. Five hundred and fifty-four million people no longer require MDA, representing a 38% reduction in the global population at risk of infection. The GPELF thus represents one of the most cost-effective public health interventions in the world [9].

Ghana is endemic for LF, with the disease present in 98/216 districts. Since 2001 the country has implemented the GPELF strategy, with significant progress made in the control of the disease [10]. However, there are challenges to the elimination programme. Despite several years of MDA, transmission still persists in 17 of 98 endemic districts [11]. In Ghana, 100% geographical coverage for MDA was reached in 2006 [10]. As such, districts with persistent transmission have received between 13 and 19 rounds of MDA. The current concerns relate to the inability to attain optimum treatment coverage [12], inaccurate reported data [13] and socio-cultural and health system challenges [14]. These point to the existence of a number of individuals who may be harboring infection, but go untreated. In this context, we describe untreated individuals as: people who refuse to take the MDA drugs for fear of side effects; people who simply refuse the treatment; people who missed the opportunity for treatment due to travel or other commercial activities; people who are not offered treatment due the inability of community drug distributors (CDDs) to reach them, migrant populations (e.g. cattle herdsmen who though are present in a district are not resident in any particular community); and people who are excluded from treatment because they are severely ill, pregnant, within the first week of lactation or too young. With the exception of this final group of individuals, who are considered ineligible to receive treatment and hence are appropriately ‘untreated’, all the other groups of individuals mentioned above should be treated. Nonetheless, it is not the current programme practice to identify and better target these untreated groups. Consequently, the programme is therefore stuck repeating MDA *ad nauseam* without directly addressing these untreated populations, some of whom may be repeatedly missed each round. New strategies are, therefore, required to address some of these challenges to LF elimination as a public health problem.

**Fig 1:**
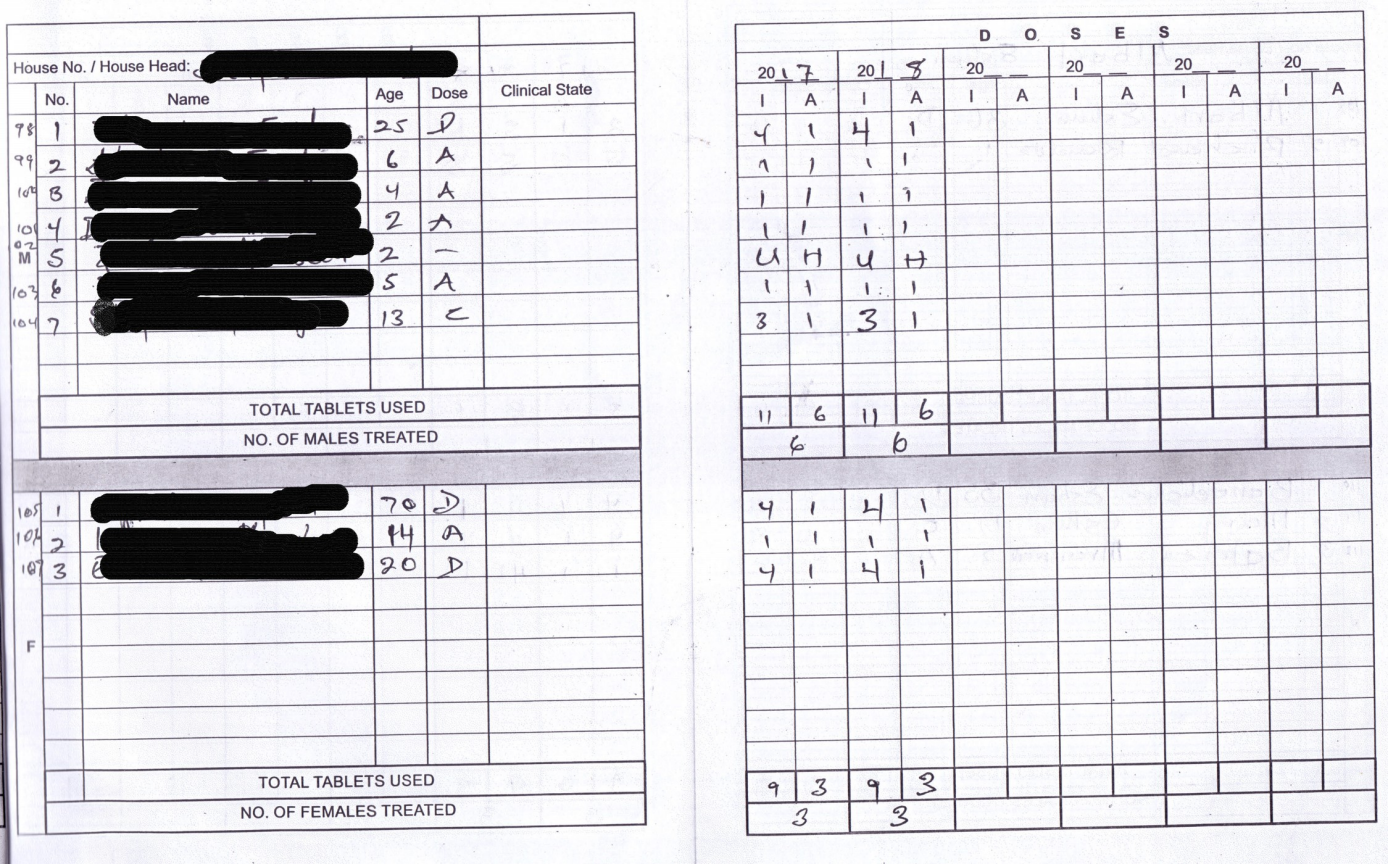
An example of an MDA register from an LF endemic community. UH: Under Height. The letters A–D refer to the ivermectin dosage where A = 1 tablet, B = 2 tablets, C = 3 tablets and D = 4 tablets. Some CDDs used the letters while others used the numbers for the tablets given.

LF MDA activities rely greatly on community registers (Fig 1) for drug distribution and reporting purposes. These registers are developed by the Ghana Health Service NTD programme and are filled by CDDs who enumerate community members, their age, gender, house numbers/names, and their participation (or lack of) in MDAs. Summary sheets in the registers make it easy to determine the number of individuals who missed treatment for various reasons. These registers are used in all communities in LF endemic districts. Prior to each MDA, cascade trainings on the data recording and reporting are undertaken from the regional through to the district and sub-district levels. Unfortunately, these MDA registers have not been fully explored, besides the collection, aggregation and reporting of data at the district level for onward transmission to the regional and national levels. This represents a global under-utilization of detailed records in the MDA registers by NTD programmes. The aim of this study was analyze the data (2016–2018) in LF registers, to verify the reported treatment coverage and characterize individuals’ compliance to MDA in selected communities.

## Methods

### Ethics statement

The study was carried out as part of a larger implementation research project titled “Community-based trial of annual versus biannual single-dose ivermectin plus albendazole against *Wuchereria bancrofti* infection in human and mosquito populations. Approval for the study was received from the Ghana Health Service Ethics Review Committee (GHS-ERC: 04112/2016) and the NMIMR IRB (CPN 062/16-17) with Federal Wide Assurance Registration (FWA 00001824). Community consent was sought for the study during meetings at which all community members are invited, at the instance of the chiefs and elders (community leaders). At these meetings, the aims of the study, procedures, risks, and benefits were explained to community leaders and members, and all concerns addressed. Written informed consent was received from all study participants.

### Study area and communities

This study was undertaken as part of monitoring surveys for the trial assessing the impact of annual versus biannual MDA with ivermectin and albendazole [15]. This trial, which started in 2017, involves 18 LF endemic communities in three districts (Ahanta West, Nzema East and Ellembele Districts) in the Western Region of Ghana. These districts have so far received between 16 and 19 rounds of MDA. In this paper, we present data for 10 villages (4 in Ahanta West, 3 in Nzema East and 3 in Ellembelle) for which it was possible to estimate MDA coverage from the registers. At the time of the data review in 2018, control communities had not received their second MDA, and therefore had no records in the registers. Thus, they were excluded from the analysis.

In the study communities, farming and fishing are the main occupations of the inhabitants. However, small scale mining and trading also contribute to the economic development of some residents. The Ahanta West district started MDA in 2000, before Nzema East and Ellembele districts in 2002. An epidemiological survey conducted in 2014 in an endemic community each in the Nzema East and Ahanta West district showed an LF (ICT) prevalence of between 8.2% and 23.5%, respectively. More detailed baseline surveys conducted as part of the above mentioned trial in 2017 estimate the mean antigen prevalence at 8.3% (95% CI: 6.9–9.9), with an estimated mean microfilaria prevalence of 1.2% [16]. As such, the districts are considered to be hotspot areas, having persistent infection despite receiving at least 16 years of MDA.

### Review of MDA registers

The registers make provision for recording the reasons for not taking part in the MDA. However, this is usually not recorded in all cases and communities. Thus, a simple questionnaire (S1 File) was developed to cross-check the participation/compliance of study participants in the 2017 MDA. As the MDA was conducted in late 2017, the questionnaire was administered in 2018. The questionnaire was administered to participants registered in the trial, and recorded the participation in the 2017 MDA, the reasons for not taking part in the MDA, adverse events (AEs) experienced, what was done for the AEs, and willingness to participate in subsequent MDAs. These data were collected as part of the above-mentioned trial to enhance the engagement with the participants and communities in order to improve future coverage rates.

In order to monitor the MDA coverage rates in the study districts, field activities were carried out to assess the coverage reported in the registers. Registers for the 10 communities were obtained from the district and the data for each community was entered into a Microsoft Excel spreadsheet. From this, the total population, population under 5 years, gender proportions and MDA target populations were determined for each year (2016–2018) and community. The number of people who participated or missed each MDA was also determined.

### Data analysis

In analysing the non-compliance, the data was combined for all communities, since they share similar characteristics. The target population eligible for MDA, i.e. population 5 years or above, was determined based on the ages recorded in the registers. The data was analyzed using MedCalc Software (Version 18.6). The verified coverage was determined as the number of eligible individuals receiving treatment (i.e. population 5 years and above that received treatment) over the number of eligible individuals requiring treatment (i.e. population 5 years and above in the community), expressed as a percentage, with 95% confidence intervals. The Graphs were drawn in Microsoft Excel.

For each community the reported and verified coverages were used to compute a verification factor (VF), as indicated in the data quality analysis tool, developed to verify the quality of reported data quantitatively [17]. The VF is computed as the ratio of verified coverage to the reported coverage, expressed as a percentage. VF values above 100% indicate under reporting, whiles values below 100% indicate over reporting. VFs between 95–105% were considered high-quality reporting. Values below 90% and above 110% were considered poor quality reporting.

## Results

There was a total of 5,538 inhabitants (2,946 females and 2,592 males) recorded in the ten community registers. The age of the population in the registers ranged from 1 month to 96 years. The population under 5 years was 975 (17.59%), leaving an MDA target population of 4,564 (82.41%). The population receiving treatment was 25.61%, 86.79% and 85.47% in 2016, 2017 and 2018 respectively.

**Fig 2:**
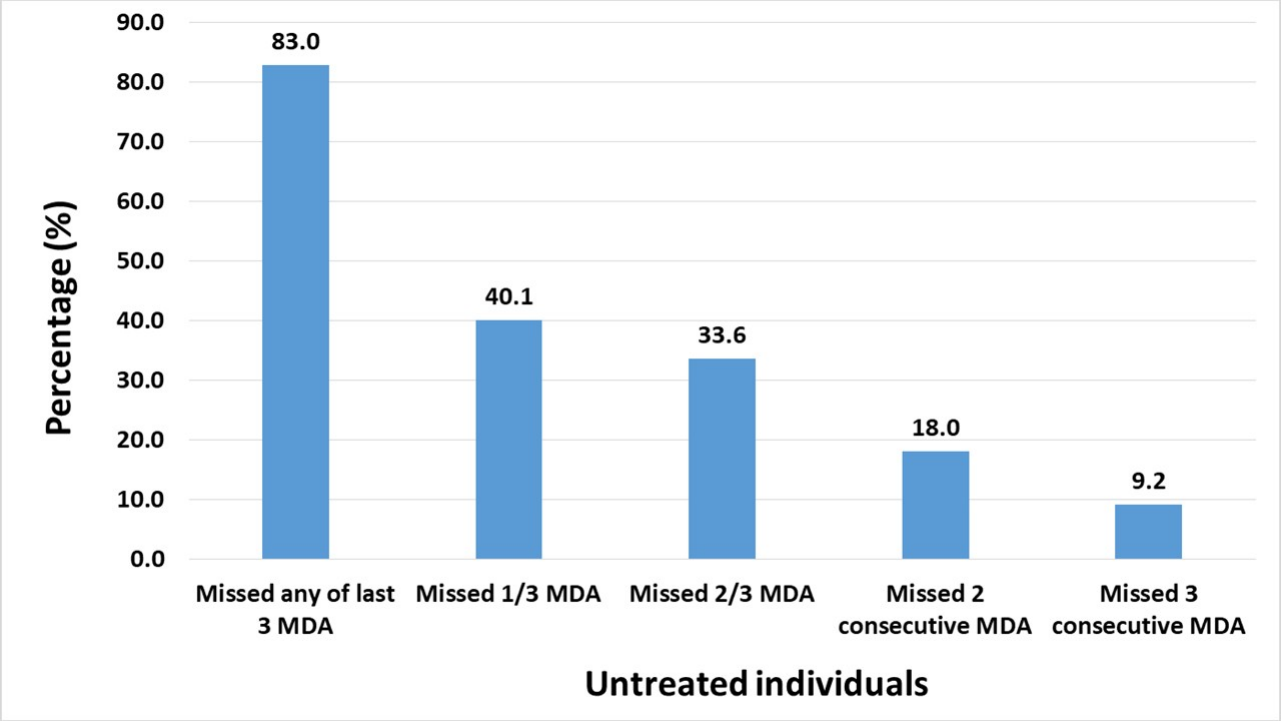
Proportion of untreated individuals observed through review of MDA registers from 2016–2018.

**Fig 3:**
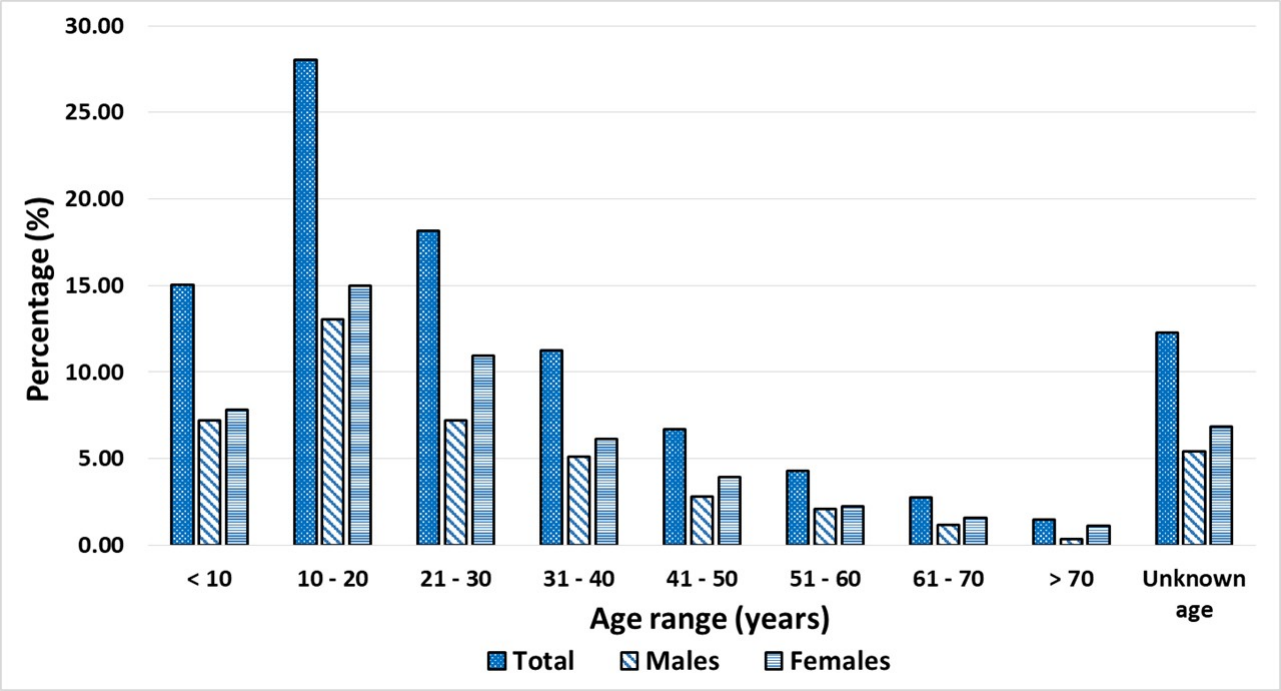
Age and gender distribution of untreated individuals observed in the MDA registers (2016–2018).

Eighty-three percent of the population missed at least one MDA in the last 3 years; 40.1% of individuals failed to take part in just one MDA, while 9.2% missed all previous 3 MDAs (Fig 2). In terms of the age of untreated individuals, the majority were between 10–30 years, with the distribution skewed towards the younger ages (Fig 3). More females were untreated compared to males, even when pregnancies were removed from the numbers untreated. There were 419 persistently untreated individuals (i.e. individuals who missed all three rounds of treatment) identified in the registers (Table 1). The majority of these were females (55.1%) and in the 10 to 30-year old age group.

**Table 1:** Details of persistently untreated individuals (missed three consecutive treatments) observed in the MDA registers (2016–2018).

	Males		Females		Total	
Age	Number	Percentage	Number	Percentage	Number	Percentage
**< 10**	10	5.3	16	6.9	26	6.2
**10–20**	46	24.5	44	19.0	90	21.5
**21–30**	36	19.1	41	17.7	77	18.4
**31–40**	15	8.0	20	8.7	35	8.4
**41–50**	11	5.9	18	7.8	29	6.9
**51–60**	9	4.8	5	2.2	14	3.3
**61–70**	8	4.3	2	0.9	10	2.4
**> 70**	1	0.5	7	3.0	8	1.9
**Unknown age**	52	27.7	78	33.8	130	31.0
**Total**	188	44.9	231	55.1	419	100.0

The results of the questionnaire assessment indicated that, out of 901 people surveyed, 13.8% (124/901) reported not participating in the 2017 MDA. Of these, the reasons for not receiving treatment were: fears of adverse events—AEs (13.7%), illness (4.8%), missing treatment (2.4%), no reason (12.1%), pregnant/breastfeeding (10.5%), refusal (4.0%), absence/travel (37.1%), and being unaware of MDA (15.3%).

**Fig 4:**
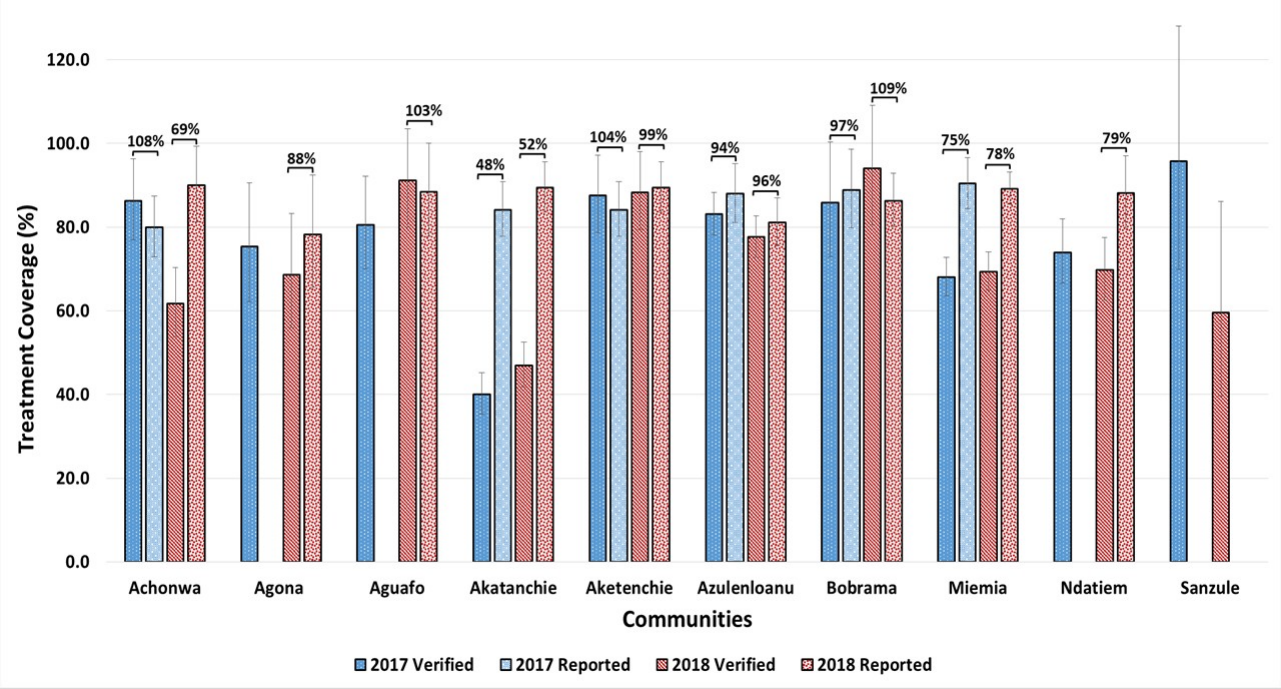
Verification of MDA coverage rates for 2017 and 2018. Error bars represent the 95% Confidence Intervals. The 2017 reported coverage for Agona, Aguafo and Sanzule, as well as the 2018 reported coverage for Sanzule were not found in the district databases. The values above the brackets indicate the verification factors between the verified and reported coverages, for the different years for each community. Values between 95 and 105 indicate high quality reporting, while values below 90 and above 110 are indicative of poor-quality reporting. Values <90 indicate over reporting and values >110 indicate under reporting. Achonwa, Agona, Aguafo and Ndatiem belong to the Nzema east District. Akatanchie, Aketenchie and Miemia belong to the Ahanta West District. Azulenloanu, Bobrama, and Sanzule are in the Ellembele District.

For each community, the data in the registers were used to verify the treatment coverage for the years 2017 and 2018, and reviewed against the reported coverage obtained from the district (Fig 4). The verification of treatment coverage began in 2017, coinciding with the start of the trial to ensure the effectiveness of the community sensitization and compliance to the MDA. Data verification of the 2016 round was not possible as coverage data for many communities was not easily accessible from the districts, due to improper storage or lack of back-up of electronic data. For many communities, the reported coverage was higher than the verified coverage. However, significant differences (p < 0.05) between the verified and reported coverages were only observed in Achonwa (2018), Akatenchie (2017 and 2018), Miemia (2017 and 2018) and Ndatiem (2018). The assessment of the VFs indicated accurate coverage reporting in only 33.3% of the cases.

## Discussion

Understanding the role of individuals’ participation in MDA and its impact on achieving the elimination of LF as a public health problem is important to address the challenges to elimination in endemic settings. This study aimed to analyse the data presented in MDA registers to assess the coverage and individuals’ compliance in MDA. It demonstrates the wealth of information and insight that can be obtained by reviewing MDA registers, a readily available resource that has hitherto been underutilized by NTD programs. A careful review of the registers data revealed that a substantial proportion of the population missed at least one treatment round, while nearly 10% of the population missed the last three rounds. In hotspot communities, such as these, where the district has failed to meet prevalence threshold required to stop MDA, identifying these systematically non-compliant individuals and developing strategies to encourage them to participate is imperative to future programme success.

While previous studies identified potential reasons for non-compliance in the study population [14], the observation of low participation in the younger people of working age could be linked to socio-economic activities. The majority of the population is made up of farmers and fishermen, whose major economic activities take place in the early morning, and sometimes involve children below the ages of 18 years. When community members were asked to provide their perspective on the persistent transmission in their district, they indicated that it was the responsibility of the CDDs to address the treatment challenges linked to socio-economic activities by revising when and how the drugs are distributed [14]. However, the community members indicated that this is not done and the CDDs reportedly start the distribution of the drugs at their own convenience. In this study we also observed that proportionately fewer females were treated than males, and we are unable to state with certainty the reasons behind this. However, this could be linked to the role of women in the social context, being responsible for taking care of the home, cooking, fetching water, in addition to helping in economic activities. Finally, the evaluation of coverage rates also confirms the results of earlier studies that revealed inaccuracies in the coverage data reported [13].

The review of the registers led to the identification of problems in the way MDA was recorded. In the registers, it could be observed that in some cases, children less than 5 years old were recorded as having received treatment (Fig 1), going against the ivermectin treatment guidelines [18, 19]. It appeared the dosage pole was used on all individuals, irrespective of age, and treatment given so long as they met the minimum height requirement. However, it was also observed that most of the treatment of underage people occurred in particular communities. In other cases, the ages of individuals in the communities were not registered. While treatment dosage is based on height and may pose no obstacles to individuals being treated, it presents difficulties when it comes to the review of registers, especially in verifying that underage children were not treated. These individuals were labelled as “unknown age” in all age-dependent analysis (Fig 2 and Table 1). Another challenge relating to the age of the participants, is the inability to determine the year in which it was recorded. While the ages are recorded in years, or months for infants, the registers make no provision for recording the dates of birth. As such, it is impossible to determine with certainty if an individual’s age was recorded the year the register was put into use, or the year the register was updated with the details of new community members. It is important to note that according to programme guidelines, registers must be updated every year immediately before MDA. The registers also provide space to record five consecutive years of MDA. Thus, while new members may be added every year, the ages of old members are not updated accordingly. Perhaps, the use of the year of birth instead of age of participants would be more valuable. While, it has been observed that some community members, especially older rural dwellers, do not know their date of birth and use important events to describe when they were born, approximate birth years could be used in such cases. Finally, the sections of the register aimed at recording the participation in the MDA are not filled to reflect the start year of MDA for the individual, such that an individual migrating to the community and recorded in the register in 2017 would not be expected to take part in the 2016 MDA. Therefore, this may affect the non-compliance rates provided in Figs 1 and 2. The above challenges present an opportunity to update new registers.

The registers also reveal issues on the treatment dosage, as different dosages were sometimes reported for the same individuals over the years. While increasing dosages may be expected from one year to another (as participants grow in stature), decreasing dosages are problematic and may reveal the difficulties of CDDs in accurately using the dosage poles. Finally, in comparing the verified coverages to the reported coverages, it was observed that the records of some communities were not included in the data used to estimate the coverage for the district. The review of the MDA registers therefore provides an opportunity to improve on the training given to CDDs and supervisors.

Despite the challenges noted above, the registers do offer opportunities to address the challenges relating to low programme coverage and non-compliance. Ensuring that no one is left behind in attaining universal health coverage is part of the Geneva commitment signed at the end of the NTD summit in 2017 [20], and will require making changes to programme activities and dedicating more resources to improving MDA implementation and social mobilization [21]. Through a review of the register after each MDA, untreated individuals could be identified. The registers also enable CDDs to record the reasons for missed treatments, even though it was observed that this was not recorded in all cases and communities. However, following our questionnaire assessments, individuals who missed treatment for reasons of illness, no reason, travel or being unaware of treatment can be classified as “not-reached”. On the other hand, individuals who plainly refuse MDA or for fears of AEs can be classified as “refusals”. Based on these classifications, untreated individuals can be targeted using two possible strategies implemented in an extended MDA campaign, i.e. beyond the two weeks during which MDAs are implemented. The MDA register makes it possible to identify individuals “not reached”, through the status definitions provided in the register. These individuals can be engaged and treated in an “Engage & Treat” strategy, since their reason for missing treatment is a result of absence or illness at the time of the MDA. In this strategy, individuals “not-reached” can be followed up by the CDDs after the MDA, and engaged/sensitized to receive treatment. Sensitization guidelines can be developed for the engagement process and provided to the CDDs. This will also enhance compliance in future MDAs. “Refusals” on the other hand may require a different strategy, such as “Test & Treat” [22–24], since their reason may be fear of AEs, or assumed non-infection/ not-at-risk. In the “Test & Treat” strategy, CDDs may need to probe further to identify the true reason for non-compliance. For individuals still unwilling to comply with treatment, testing with the Filariasis Test Strip could be offered by a trained community health personnel, using test kits provided by the NTD programme; individuals testing negative would be allowed to abstain from treatment, while those testing positives would then be further engaged to receive treatment. It will be important to work with the social science community to train CDDs on appropriate engagement strategies to encourage greater MDA compliance. These engagement strategies will be particularly important in the event that some individuals may initially reject both testing and treatment.

Where MDA registers exist, we believe that careful review of these registers, combined with targeted programmatic actions, such as ‘Engage & Treat’ and ‘Test & Treat’, is a useful way to address challenges related to low MDA coverage. This approach should be explored further at the community and implementation unit levels, particularly in sites where coverage is consistently low. It is worth noting that these strategies may come with the additional challenge of how best to report the data after the MDA. Also, community members may miss treatment during the main MDA, knowing they can always receive treatment at another time in the year. Thus, when, where and how to implement these strategies are all important decisions that will need to be made at the country level. Nonetheless, the “Engage & Treat” or “Test & Treat” strategies following a review of registers may represent programmatic opportunities for identifying and targeting untreated individuals and can be used to ensure that as much of the population is treated as possible.

It is important to note that a couple of tools have been developed in the attempt to improve treatment coverage. These include the coverage evaluation surveys, data quality assessments (DQA) and supervisor’s coverage tool (SCT)[25] each of which have advantages and disadvantages. A primary aim of the coverage evaluation survey is to validate the reported coverage at the implementation unit level using a rigorous sampling methodology conducted by a survey team that is external to the programme. Consequently, this tool has considerable time and cost implications that cannot be ignored. DQAs verify reported data and assess the capacity of data management and reporting systems. However, these occur at the implementation or national level and can be expensive. The SCT, on the other hand, aims to classify coverage as above or below the target threshold. It is implemented in near real-time (within 2 weeks of MDA) at the lowest unit of intervention (community) or supervisory area (sub-district), by internal personnel, and is cheaper than both the coverage surveys and DQAs. However, while the SCT is aimed at classifying whether the minimum coverage requirements are met, during the period of the MDA, it does not consider the characteristics of those who went untreated, nor does it provide a measure of systematic non-compliance. For example, if the same 65% of the population takes part in MDA every year, each MDA would technically be classified as effective (having achieved the minimum coverage target for LF); however, the remaining 35% of the population that is routinely untreated may harbor infection. The proposed strategies presented herein aim to target these individuals that may be acting as reservoirs of infection within the community, and are systematically missed during MDA.

The strategy of identifying and treating individuals not treated during the MDA campaign has been used in the Democratic Republic of Congo [26]. Presumably, this will lead to achieving coverage rates above the recommended 65% levels. Modelling studies [27–29] have shown that the number of MDA rounds required to interrupt transmission decreases with higher coverage levels and particularly when the proportion of those who systematically fail to comply is minimized. Thus, achieving the interruption of transmission and elimination may be faster if MDA coverage and compliance is high.

Currently, CDDs have two weeks to implement MDA, which may not be sufficient time to identify and cover every eligible member of the community. The register review strategy proposed here changes the current paradigm; treatment of unreached or non-compliant individuals is intended to be a continuous process after MDA, till such a time when CDDs have identified and offered the drugs to all available community members. A disadvantage of the strategy is the lag-time added after the MDA in order to ensure maximum coverage. There may also be the tendency for individuals to assume treatment will be available after MDA or that they can opt for testing before treatment, resulting in low coverage rates for MDA. Further, this may not be sustainable over time and is not intended for implementation in all areas but only in districts where current strategies are insufficient at suppressing transmission. In this regard, social science methods will be useful to understand who non-compliant individuals are more broadly and what would motivate them to comply in the future. This information could then be used to adapt the social mobilization and treatment delivery strategies of the programme and hopefully lead to an increase in compliance during future MDA rounds.

The register review strategy may also present opportunities to collaborate with modellers to integrate LF prevalence information from non-compliant individuals into transmission models and better understand how this impacts the elimination goals. Additionally, non-compliant individuals who test positive for filarial antigens could be offered a more effective treatment regimen such as the ivermectin + DEC + albendazole (IDA) combination, a single dose of which has been shown to be highly effective at clearing microfilaremia [30] and obviates the requirement to provide 5 rounds of annual treatments. However, further evaluations and prior testing would be required, as IDA treatment cannot be offered to individuals infected with onchocerciasis or loa loa [7].

In conclusion, this study revealed the programmatic gains that can be achieved by reviewing MDA registers, which are a straight forward and readily available source of information. Besides their standard use for recording and reporting MDA data, registers offer a unique opportunity to identify specific individuals, as well as the general demographics of people, who do not receive treatment and provides one of the only means for identifying systematically non-compliant individuals [26, 31–34]. It is important to acknowledge that there will be some training, cost and time requirements in order to employ the strategies proposed here and that the CDDs and supervisors will play a critical role in the process. Finally, it will be important to understand whether the incremental increases in coverage brought about by this approach merit the time and financial investments required. Ensuring no one is left behind will require the collective effort of the NTD community and a willingness to change policy guidelines, moving resources to target challenging areas and increasing the cost-per-treatment as elimination programmes exhaust the economies of scale and scope and move toward harder-to-reach populations [21, 35].

## Supporting information

S1 File Questionnaire guide for assessing participation in MDA. (DOCX)
