# Supplementary material for: Review of MDA registers for Lymphatic Filariasis: Findings, and potential uses in addressing the endgame elimination challenges
Source: PLoS Negl Trop Dis. 2020 May 14;14(5):e0008306. doi: 10.1371/journal.pntd.0008306 (PMC7252669; doi:10.1371/journal.pntd.0008306)
Supplement: S1 File — (DOCX) [file pntd.0008306.s001.docx]

**Treatment Monitoring Survey Guide:**

Date:

Participant ID (From study register):

District (From study register):

Community (From study register):

House No (From study register):

Age (From study register):

Gender (From study register):

**Q1:** Did you receive treatment in 2017? (Yes/No)

**Q2:** If the answer to Q1 is no, why not?

**Q3:** Did you receive treatment in 2018? (Yes/No)

**Q4:** If the answer to Q3 is no, why not

**Q5:** Did you experience any AEs after the treatment?

**Q6:** If the answer yes, describe what you experienced

**Q7:** Will you recommend the drugs to anyone?

**Q8:** Given your adverse reactions will you still recommend the drugs to anybody?

**Q9:** Any remarks?

*THANK YOU VERY MUCH FOR PARTICIPATING IN THE INTERVIEW*
